# Complete Genome Sequence of Avian Coronavirus Strain D274

**DOI:** 10.1128/MRA.01003-18

**Published:** 2018-08-30

**Authors:** Paulo E. Brandão, Aline S. Hora, Sheila O. S. Silva, Mikael Berg, Sueli A. Taniwaki

**Affiliations:** a Department of Preventive Veterinary Medicine and Animal Health, School of Veterinary Medicine, University of São Paulo, São Paulo, Brazil; b School of Veterinary Medicine, Federal University of Uberlândia, Uberlândia, MG, Brazil; c Department of Biomedical Sciences and Veterinary Public Health, Section of Virology, Swedish University of Agricultural Sciences, Uppsala, Sweden; Louisiana State University

## Abstract

Avian coronavirus, the causative agent of avian infectious bronchitis, occurs as multiple genotypes and lineages, and full genomes are not available for the majority of them. This paper reports the (previously unknown) complete genome sequence of strain D274 of this virus (27,599 nucleotides), isolated from chickens in The Netherlands in 1979.

## ANNOUNCEMENT

A plethora of types of avian infectious bronchitis virus (IBV) (Nidovirales: Coronaviridae: Coronavirinae: Gammacoronavirus: Avian coronavirus [AvCoV]) strains are used in live and inactivated vaccine formulations worldwide to prevent infection with or lessen the symptoms caused by the diverse number of field strains of the virus in chickens (1).

IBV occurs as 6 genotypes with a total of 34 lineages based on the spike S gene and is involved in multisystemic, highly contagious infections of chickens (1, 2). The IBV positive-sense, single-stranded 5ʹ capped RNA genome with circa 27 kb codes for the replicase complex (open reading frame 1 [ORF1]); the four structural proteins spike S, envelope protein E, membrane protein M, and nucleocapsid protein N; and accessory proteins 3a, 3b, 5a, and 5b, with an untranslated region (UTR) at both the 5ʹ and 3ʹ ends of the genome (3, 4).

The Gammacoronavirus/AvCoV/chicken/Netherlands/D274/1979 IBV strain (lineage GI-12) was isolated from tissue samples of chickens collected in 1979 in The Netherlands (5) and is used as an inactivated intramuscular vaccine strain in several countries, including Brazil. An aliquot of a D274 strain used in an inactivated vaccine was clarified at 12,000 × *g*/15 min at 4°C and then filtered (0.45 µm) and treated with DNase/RNase, and the next total RNA was purified using TRIzol reagent (Life Technologies) and an RNeasy minikit (Qiagen). SuperScript III and Klenow exo-DNA polymerase (Life Technologies) were used to produce double-stranded cDNAs (ds-cDNAs) using total RNA containing 5,000 copies/µl of IBV genome as measured with a quantitative PCR (qPCR) modified from reference 6.

The libraries and sequencing kits used were by Illumina (Nextera XT Index and Nextera XT DNA), and reads were obtained with a NextSeq 500 system (Illumina) using the NextSeq 500 mid-output v2 kit (150 bp). Out of 75,931,144 total reads, 67,015,998 reads were mapped using CLC Genomics Workbench v11.0.1 (Qiagen) with AvCoV isolate 23/2013 (GenBank accession number KX258195) as a reference, as *de novo* assembly did not produce a complete and viable genome using the same software.

The D274 genome is 27,599 nucleotides (nt) long [poly(A) tail included] and annotated in the order 5ʹ UTR and leader RNA (nt 1 to 528), ORF1a (nt 529 to 12327), and ORF1ab (nt 529 to 20360) with a ribosomal frameshift, followed by the spike S (nt 20311 to 23802), 3a (nt 23802 to 23975), 3b (nt 23975 to 24169), envelope E (nt 24150 to 24473), membrane M (nt 24442 to 25122), 5a (nt 25481 to 25678), 5b (nt 25675 to 25923), and nucleocapsid N (nt 25866 to 27095) genes and a 3ʹ UTR (nt 27096 to 27599).

Reads were used to run whole-genome low-variant detection using CLC Genomics Workbench v11.0.1 (Qiagen) (≥100× coverage, ≥10 counts, ≥5% frequency, 1% significance, and ≥20 central and neighborhood qualities) to assess the quasispecies population of D274, i.e., the population of genome variants, including a dominant sequence subjected to natural selection and drift, resulting in only 22 mutations, showing a rather high homogeneity on this D274 sample.

Spike protein sequences, easily obtained by Sanger sequencing, are widely used to speculate about vaccine protection (7), but as the immunity for IBV is not based only on the humoral response to this protein (8, 9), the availability of full genomes for vaccine strains is core to accurately predicting protection markers.

### Data availability.

The Gammacoronavirus/AvCoV/chicken/Netherlands/D274/1979 complete genome sequence is deposited in GenBank under the accession number MH021175. The version described in this paper is the first version, MH021175.1.

## References

[1] Jackwood MW 2012 Review of infectious bronchitis virus around the world. Avian Dis 56:634–641. doi:10.1637/10227-043012-Review.1. 23397833

[2] Jiang L, Zhao W, Han Z, Chen Y, Zhao Y, Sun J, Li H, Shao Y, Liu L, Liu S 2017 Genome characterization, antigenicity and pathogenicity of a novel infectious bronchitis virus type isolated from South China. Infect Genet Evol 54:437–446. doi:10.1016/j.meegid.2017.08.006. 28800976 PMC7106192

[3] Jackwood MW, Hall D, Handel A 2012 Molecular evolution and emergence of avian gammacoronaviruses. Infect Genet Evol 12:1305–1311. doi:10.1016/j.meegid.2012.05.003. 22609285 PMC7106068

[4] Cavanagh D 2007 Coronavirus avian infectious bronchitis virus. Vet Res 38:281–297. doi:10.1051/vetres:2006055. 17296157

[5] Davelaar FG, Kouwenhoven B, Burger AG 1984 Occurrence and significance of infectious bronchitis virus variant strains in egg and broiler production in The Netherlands. Vet Q 6:114–120. doi:10.1080/01652176.1984.9693924. 6091319

[6] Callison SA, Hilt DA, Boynton TO, Sample BF, Robison R, Swayne DE, Jackwood MW 2006 Development and evaluation of a real-time TaqMan RT-PCR assay for the detection of infectious bronchitis virus from infected chickens. J Virol Methods 138:60–65. doi:10.1016/j.jviromet.2006.07.018. 16934878 PMC7112890

[7] Chacón JL, Assayag MS Jr, Revolledo L, Astolfi-Ferreira CS, Vejarano MP, Jones RC, Piantino Ferreira AJ 2014 Pathogenicity and molecular characteristics of infectious bronchitis virus (IBV) strains isolated from broilers showing diarrhoea and respiratory disease. Br Poult Sci 55:271–283. doi:10.1080/00071668.2014.903558. 24678626

[8] Han Z, Zhao F, Shao Y, Liu X, Kong X, Song Y, Liu S 2013 Fine level epitope mapping and conservation analysis of two novel linear B-cell epitopes of the avian infectious bronchitis coronavirus nucleocapsid protein. Virus Res 171:54–64. doi:10.1016/j.virusres.2012.10.028. 23123213 PMC7114416

[9] Okino CH, dos Santos IL, Fernando FS, Alessi AC, Wang X, Montassier HJ 2014 Inflammatory and cell-mediated immune responses in the respiratory tract of chickens to infection with avian infectious bronchitis virus. Viral Immunol 27:383–391. doi:10.1089/vim.2014.0054. 25105981

